# Social Media Use and Misinformation Among Asian Americans During COVID-19

**DOI:** 10.3389/fpubh.2021.764681

**Published:** 2022-01-14

**Authors:** Stella K. Chong, Shahmir H. Ali, Lan N. Ðoàn, Stella S. Yi, Chau Trinh-Shevrin, Simona C. Kwon

**Affiliations:** ^1^Department of Population Health, Section for Health Equity, New York University Grossman School of Medicine, New York, NY, United States; ^2^Department of Social and Behavioral Sciences, New York University School of Global Public Health, New York, NY, United States

**Keywords:** Asian American, social media, misinformation, infodemic, COVID-19, mass media, health misinformation, health disparity

## Abstract

Social media has been crucial for seeking and communicating COVID-19 information. However, social media has also promulgated misinformation, which is particularly concerning among Asian Americans who may rely on in-language information and utilize social media platforms to connect to Asia-based networks. There is limited literature examining social media use for COVID-19 information and the subsequent impact of misinformation on health behaviors among Asian Americans. This perspective reviews recent research, news, and gray literature to examine the dissemination of COVID-19 misinformation on social media platforms to Chinese, Korean, Vietnamese, and South Asian Americans. We discuss the linkage of COVID-19 misinformation to health behaviors, with emphasis on COVID-19 vaccine misinformation and vaccine decision-making in Asian American communities. We then discuss community- and research-driven responses to investigate misinformation during the pandemic. Lastly, we propose recommendations to mitigate misinformation and address the COVID-19 infodemic among Asian Americans.

## Introduction

During public health emergencies like the 2003 SARS pandemic, 2009 H1N1 swine flu pandemic, 2016 Ebola epidemic, and current COVID-19 pandemic (1–4), the internet and social media serve as hubs for the growth and rapid dissemination of information and misinformation globally, often contributing to confusion in public health campaigns. Misinformation is false or inaccurate information that is not supported by scientific evidence and shared without intent to cause harm, and includes conspiracy theories, disinformation, and mal-information (5, 6). Misinformation is often conflated with colloquially used terminology such as fake news, rumor, urban legends, spam, and trolling (6, 7). This overabundance of information and misinformation contributes to the growing infodemic, where the availability of false or misleading information online and offline creates an environment that can undermine public health responses during a crisis (8). Misinformation has been shown to negatively impact people's COVID-19 health protective behaviors, including spending limited time outside of homes, practicing social distance measures, and washing hands per public health guidance (9). COVID-19 misinformation, specifically the erroneous labeling of COVID-19 as the “Chinese Virus” on social media (10) has led to social discrimination on Asian American communities, contributing to increasing anti-Asian sentiment and anti-Asian hate crimes across the nation.

Asian American social networks are distinct from other communities. These networks may include family in other Asian countries and include dissemination of non-English information on social media. However, there is a paucity in literature examining use of social media for COVID-19 information among Asian Americans, types of misinformation disseminated on social media in these networks, and subsequently the impact of such information on Asian Americans' health behaviors. We conducted a narrative review to examine the use of social media platforms to disseminate COVID-19 misinformation across Chinese, Korean, Vietnamese, and South Asian Americans, four of the largest Asian subgroups in the United States (U.S.). We discuss the linkage of COVID-19 misinformation to health behaviors, with emphasis on misinformation about COVID-19 vaccines and vaccination confidence, as well as community-driven responses to address COVID-19 misinformation among Asian Americans. Lastly, we propose recommendations to mitigate misinformation and address the infodemic in Asian American communities.

### COVID-19 Misinformation and Social Media

Health misinformation is prevalent across various social media platforms. A systematic review of 69 studies identified Twitter, YouTube, and Facebook as the most common social media platforms for health information dissemination, while less popular platforms included WhatsApp, Pinterest, Tumblr, BK, and Myspace (11). There are six main categories of health misinformation topics including pandemics, vaccines (e.g., antivaccination, side effects of vaccines), medical treatments, non-communicable diseases (e.g., cancer, diabetes, epilepsy), eating disorders, and drugs or smoking (11). Health misinformation poses a public health challenge due to its adverse influence on health behaviors, including panic buying and disregarding health guidance from public health officials (e.g., stay-at-home orders, handwashing, social distancing) (9, 12, 13).

Researchers reported that social media was nationally the third most common information source for COVID-19 information and misinformation (14). Similarly, a recently published scoping review examining the role of social media during the COVID-19 pandemic identified 12 articles that discussed the role of COVID-19 misinformation augmenting the global infodemic (15). The ongoing infodemic has spread rumors and disinformation on social media related to COVID-19 virus etiology, prevention, treatment, interventions, and vaccines (16, 17). COVID-19 misinformation is challenging to address and document given its pervasiveness across various social media platforms that are highly utilized and trusted by the global community (18). Greater exposure to misinformation is associated with problematic COVID-19 beliefs, poorer COVID-19-related knowledge, and less preventative behaviors (13). Relatedly, belief in COVID-19 conspiracy theories was found to be associated with lower COVID-19 preventative behaviors (9). Misinformation about COVID-19 may also influence specific health behaviors (e.g., vaccine hesitancy) (9, 19).

### Social Media Utilization Among Asian Americans

Asian Americans, who comprise 5.5 percent of the U.S. population (20), are leading users of internet and mobile technologies, evidenced through higher ownership of smartphones, laptops, and wireless networks (21, 22). Approximately 94 percent of Asian American households own a smartphone in the US, which increases access to the internet and social media for entertainment, information seeking, and social connectedness (22). In particular, Asian Americans (many of whom are first generation, foreign-born immigrants) utilize the internet and social media to seek and receive up-to-date health information (23, 24). According to the Pew Research Center (25), approximately two-third of Asian Americans speak a language other than English at home, with Chinese (34%) being the most spoken Asian language, followed by Hindi (13%), Tagalog (9%), and Vietnamese (4%). Social media allows for cross-national social connectedness to family and friends in home countries (22), and the efficacious transfer of health, political, economic-related information in native languages. Importantly, for limited English proficient (LEP) Asian Americans, the use of mainstream English-speaking social media platforms may not be appropriate. To mitigate language barriers and receive up-to-date health information, many Asian Americans tend to use social media platforms that enable communication in their native languages. As such, preference for social media platforms among Asian Americans parallels the diversity of Asian American ethnicities, languages, and generation (see Table 1).

**Table 1:** Preferred digital/social media platforms by Asian American subgroup.

**Asian American subgroup**	**Preferred digital/social media platforms**
Chinese Americans	(China) WeChat (26, 27)
	(Taiwan) Line (26, 27)
	(Hong Kong) WhatsApp (26, 27)
Vietnamese Americans	Facebook and YouTube (26, 27)
Korean Americans	KaKaoTalk (26, 27)
Japanese Americans	Line (26, 27)
Asian Indian Americans	WhatsApp (26, 27)

Despite the prevalent use of social media among Asian Americans, there has been limited research on Asian Americans' pattern of social media use by age, gender, and socioeconomic status (28). Frequently, Asian Americans are not included in national datasets on social media use and engagement. For instance, the Pew Research Center does not report data on Asian Americans in its research on social media use in the U.S. Similarly, detailed race/ethnicity was not reported for “Americans” in an international survey that examined use of social media for health information among Americans, Koreans, and Hong Kongers (29). In the context of the COVID-19 pandemic, there has been no research investigating Asian Americans' use of social media for COVID-19-related information. While recent research has investigated sociodemographic predictors of U.S. adults' use and trust of COVID-19-related information sources (14), researchers have yet to examine race as a factor, limiting what we know about Asian Americans' use of social media for COVID-19 related-information. However, a recent survey of Asian American young adults from March 2021 reported participants used Facebook Messenger (61%) and Instagram (57%) most often with siblings and cousins and WhatsApp (29%) with parents^1^. Use of social media platforms also varied by Asian subgroup, gender and country of birth^1^. Preference of specific social media platforms may also differ between older and younger generations of Asian Americans. However, there may be in-language social media platforms such as WeChat, KaKaoTalk, and Line that are shared across generations and promote the circulation of information across geographic and generational boundaries.

### COVID-19 Misinformation by Asian American Subgroups

Amidst COVID-19-related uncertainty and fears, Asian Americans are turning to the internet and social media for the latest updates on the pandemic, such as confirmed COVID-19 cases, government-issued COVID-19 policies and guidelines, and COVID-19-related health information (e.g., COVID-19 prevention measures, personal protective equipment (PPE), COVID-19 symptoms, diagnosis, and treatment) (30, 31), which have been shown to positively influence and improve public health behavioral strategies against COVID-19 (1). Asian Americans' pattern of utilizing social media and other online sources for information during the COVID-19 pandemic is analogous to the use of these platforms during the 2003 SARS pandemic (2), during which the Centers for Disease Control and Prevention (CDC) reported evidence of substantial misinformation that contributed to growing confusion and fear. Likewise, COVID-19 misinformation is circulating on social media platforms utilized by Asian Americans, leading to widespread misinformation across the Asian American communities in the U.S. and the Asian global diaspora. Misinformation on the internet and social media during the COVID-19 pandemic continue to heighten the mistrust, fear, and anxiety stimulated by COVID-19 pandemic and anti-Asian racism (32, 33).

Extant literature on social media, Asian Americans, and COVID-19 has focused on how contentious COVID-19 misinformation contributed to the increase in discrimination, racism, and violence against Asian Americans (10, 34), as well as how social media is used to mediate such effects (35). However, there is limited research on the content of COVID-19 misinformation dispersed across ethnic-specific social media platforms in the Asian American community. We illustrate the heterogeneity of COVID-19 misinformation across social media applications by Asian American subgroups and the potential impacts of COVID-19 misinformation on Asian American subgroups' health behaviors (see Table 2).

**Table 2:** COVID-19 misinformation on social media platforms by Asian American subgroup.

**Asian American subgroup**	**Social media platform**	**Special features**	**COVID-19 misinformation**	**Significance of social media platform in disseminating misinformation**	**(Potential) Impact(s) of misinformation**
Chinese Americans	WeChat	• Moments (朋友圈 péng you quān): private chat groups and private news feed (36). • Forwarding and sharing	Conspiracy Theories: • Origin of the SARS-CoV-2 virus as a bioweapon engineered in the U.S. in the economic and psychological warfare against China (16) • Creation of the SAR-CoV-2 virus by the Chinese government in the laboratory as part of its national biowarfare program (16) COVID-19 Prevention and Treatment: • Avoiding cold foods and beverages • Drinking hot water COVID-19 Vaccines: • Rumor that Moderna vaccine is more effective than Pfizer vaccine (37)	• WeChat is used by 19 million individuals in the United States and has emerged as the central hub for COVID-19 information and misinformation sharing in the Chinese American community, particularly among Chinese immigrants.	• Conspiracy theories are concerning as over a quarter (26%) of Americans believe that COVID-19 was released as an act of bioterrorism (14). • Vaccination hesitancy among the Chinese American community was observed in several COVID-19 vaccine webinars hosted in Mandarin and Cantonese Chinese by the NYU CSAAH where webinar attendees raised comments questioning the efficacy of the Pfizer vaccine over the Moderna vaccine. Anecdotal evidence from the community suggests that some community members contemplated delaying their vaccination to get the Moderna vaccine over the Pfizer vaccine.
Korean Americans	KaKaoTalk	• Forwarding and sharing	Spread of COVID-19: • Rumor that Korean Air flight attendant with the coronavirus had dined at a local Los Angeles (LA) Koreatown restaurant (38) COVID-19 Prevention and Treatment: • Drinking hot water at 15-min intervals (39) • Spraying salt water (40) • Consuming curry powder and garlic (40)	• KaKaoTalk has been cited as the main source of disinformation by a former staff from Korea's National Assembly Defense Committee (41).	• The impact of the rumor in the Korean American community was instrumental, leading to decline of diners and sales at Korean restaurants (38).
	YouTube			• Research on Korean-language COVID-19 related medical information on YouTube found that 37% of the 105 analyzed YouTube videos contained misinformation (42).	
Vietnamese Americans			Parallels that of the Chinese American community, such that misinformation is centered on: • Conspiracy theories on virus' origin (26) • Progression of the pandemic (26) • Prevention and treatment home remedies (26) COVID-19 vaccines: • No need to wear mask post-vaccination (43) • No need to get vaccination if one has already tested positive for COVID-19 (44) • One dose of COVID-19 vaccine is sufficient for protection (45)		• Vaccine hesitancy among community members
	YouTube (TheKingRadio and TrainMaicoUSA)	• In-language content	COVID-19 Vaccines: • Conspiracy theories of secret government (46) • Vaccine-induced COVID-19 contractions (46) • Vaccine-induced deaths (46)	• Closely tied to politics and intergenerational trauma • Red-baiting, tactic used commonly to advance specific political agendas by labeling policies and politicians as “socialist” or “communist” • According to the Progressive Vietnamese-American Organization (PIVOT), the Vietnamese community is red-baited to accept misinformation that denounces socialism and communism.	• Vaccine hesitancy among community members
	Facebook and email chains				
	Local television news channels		COVID-19 Prevention and Treatment: • Gargling salt water (47)		
South Asian American	WhatsApp and Facebook	• Direct private messaging between individuals and groups of family and friends (48) • Forwarding and sharing (48)	Role of Religion in Misinformation: • Presence of animal products in vaccines (e.g., pork-related concerns among Muslims or beef-related concerns among Hindus) (49) • Religious tensions between Muslims and Hindus (e.g., Muslims licking plates, spitting on dollar bills, hashtags such as #CoronaJihad (50) COVID-19 Prevention and Treatment: • Wearing hijab to prevent COVID-19 spread (51)		• Vaccine hesitancy among community members

### Efforts to Addressing COVID-19 Misinformation in Asian American Communities

In response to alarming COVID-19 misinformation in Asian American communities, community and public health leaders are spearheading efforts to combat the impacts of misinformation and to disseminate scientific COVID-19 information. In New York City, in-language virtual webinars were held by the NYU Center for the Study of Asian American Health (CSAAH), in partnership with community and academic partners, for Chinese, Filipino, South Asian, Arab American, and Hispanic communities, to answer questions and concerns about COVID-19. Likewise, the Filipino Young Leaders Program (FYLPRO) developed the Caretaker Program, Tayo, a virtual help desk that provides accurate and timely information on COVID-19 to the Filipino community in Tagalog and English (52). Similarly, the Asian Pacific Islander American Health Forum (APIAHF) has produced in-language videos (e.g., Simplified Chinese, Traditional Chinese, Korean, Vietnamese, and Bengali) to dispel COVID-19 vaccine misinformation in Asian American communities (53).

Nationally, the Progressive Vietnamese American Organization (PIVOT) launched the Viet Fact Check Project to release in-language information on COVID-19 vaccination, to dispel common myths, and to encourage higher uptakes of vaccination in the Vietnamese community (46). The Viet Fact Check Project is also collaborating with the University of Washington Center for an Informed Public, to examine the pattern of misinformation and media consumption in the Vietnamese community (54). Similarly, for the South Asian community, the University of California, San Francisco and California State University, East Bay are conducting a study called, Covid Associated MisinforRmation on Messaging Apps (CAROM) (55), to evaluate COVID-19 misinformation circulating in WhatsApp among South Asians.

## Recommendations

Misinformation, especially COVID-19 misinformation, has far-reaching impacts on health and wellbeing for Asian Americans and other minority groups that face existing, unique health disparities (9, 10, 13, 56). It is imperative to recognize the abundance of misinformation that exists in these communities and to identify misinformation as a public health crisis (9, 12, 13, 18). The field of public health, with its mission of prevention and education, can informatively identify misinformation and provide accurate scientific facts about COVID-19. Prior research has shown that correction is an effective measure to counter health misinformation on social media (57). In the correction approach, misinformation on social media is identified with a timely and credible explanation of why the misinformation is false, and the explanation is repeatedly reinforced through multiple corrections (57). A collaborative taskforce of public health officials and digital health communication specialists monitoring should be assembled on these in-language social media platforms to promptly identify and address COVID-19 misinformation that are available in English and Asian languages. Moreover, public health organizations can collaborate with leading social media and technology companies to efficiently detect, label, and remove COVID-19 misinformation, as well as to provide accurate public health announcements. Recently, the World Health Organization (WHO) has been collaborating with social media companies including WhatsApp, Viber, TikTok, Google, and YouTube to ensure that accurate, reliable, scientific information about COVID-19 appears first when people search for COVID-19-related information (58). However, leading in-language social media platforms like WeChat and KaKaoTalk also need to be included in this collaborative effort to stamp out COVID-19 misinformation in Asian American communities.

Identification and correction of misinformation across social media is not enough to end the COVID-19 infodemic. In concordance with the deficit hypothesis (59), public health also needs to improve eHealth literacy among the public to ensure that people are thoroughly assessing the scientific quality and accuracy of the information they encounter on social media (60). eHealth literacy is “the ability to seek, find, understand, and appraise health information from electronic sources and apply the knowledge gained to addressing or solving a health problem (61).” A cross-sectional survey conducted in China found that eHealth literacy is a significant predictor of preventative health behaviors during the COVID-19 pandemic and that eHealth literacy has more impact on health-related behaviors than knowledge (62). Findings from this study suggest that improving and targeting eHealth literacy is critical to manage the COVID-19 crisis (62). In order to improve eHealth literacy and ultimately mitigate COVID-19 misinformation among Asian Americans, in-language training and materials need to be made more available. Through these trainings and materials, Asian American community members can be provided with better tools to decipher between reliable, accurate scientific information on COVID-19 and misinformation on social media platforms. Likewise, public health officials need to improve media literacy in Asian American communities, informing and guiding community members on how to analyze and evaluate the quality of diverse media platforms, including social media, films, newspapers, television shows, etc., for accurate scientific information (63). With improved eHealth and media literacy, Asian Americans can utilize social media to seek accurate health information, which prior studies have shown health education on social media to be effective in addressing prevalent health issues such as hypertension (64), Hepatitis B (65), and obesity (66) among Asian Americans. Lastly, as Asian American is a diverse racial group, it is necessary to hold additional culturally- and linguistically-concordant virtual webinars and create educational materials tailored to addressing the unique COVID-19 misinformation experienced by all Asian American subgroups.

In addition, the public health response can utilize and leverage community health workers (CHWs), who are vital members representative of the community, to identify COVID-19 misinformation and to inform community members of ways to be aware of misinformation and provide accurate in-language information.

Furthermore, many forms of online misinformation, particularly conspiracy theories, are due to complex psychological mechanisms that need to be further examined to thoroughly combat and contain the infodemic in Asian American and other communities. People are also more likely to have strong conspiracy beliefs that undermine any scientific guidance when they are experiencing high levels of anxiety and uncertainty (67). Research has shown that conspiracy theories are influential and often inimical to health (68), which is concerning during the COVID-19 pandemic as preventative health behaviors are instrumental to abating and containing the spread of COVID-19. Recognizing the prevalence of conspiracy theories and its adverse impact on COVID-19 vaccination acceptance and uptake (69) is necessary to develop targeted strategies and interventions to encourage vaccination among low-income and communities of color that are at higher risk for COVID-19. Interdisciplinary research examining the psychology of misinformation can inform public health efforts to stop the spread of COVID-19 misinformation (70).

## Conclusion

Social media has inevitably promulgated the transnational exchange of COVID-19 misinformation during the pandemic, prompting academic and community action to combat COVID-19 misinformation on social media. We speculate that Asian Americans are likely to utilize social media to receive COVID-19-related health information because they are able to effectively communicate with family and friends, particularly due to the dearth of COVID-19 information by the federal government in diverse Asian languages. We recommend multi-sectoral efforts between public health officials, community leaders, researchers, and social media companies to identify and mitigate health misinformation on social media, as well as the inclusion of CHWs to educate community members on misinformation and provide accurate scientific information. Improving eHealth and digital literacy among Asian Americans could also aid in the evaluation of identifying misinformation and seeking trustworthy and reputable websites. More research on the psychological mechanisms behind conspiracy theories and misinformation could led to effective health promotion communication strategies and interventions that improve acceptance of scientific evidence and public health guidance needed during public health emergencies.

Research is also needed to address the origins and mediators of susceptibility to COVID-19 misinformation. For example, the exploration of how social media platforms, usage patterns, and usage of social media for health information by disaggregated Asian American subgroups in the U.S., as well as by other racial and ethnic groups is needed. Increase research efforts are necessary to quantitatively and qualitatively understand the social, economic, and health impacts of COVID-19 misinformation in Asian American communities. The consequences of misinformation on health behaviors in Asian American communities will likely span beyond the impacts of the COVID-19 infection and will require culturally- and linguistically-concordant interventions to address health disparities.

## Data Availability Statement

The original contributions presented in the study are included in the article/supplementary material, further inquiries can be directed to the corresponding author/s.

## Author Contributions

SC, SA, SY, and SK conceptualized the manuscript. SC and SA wrote the first draft of the manuscript. LÐ, SY, and SK provided critical review of the manuscript. All authors contributed to manuscript revision, read, and approved the submitted version.

## Funding

This work was supported by the National Institutes of Health (NIH), National Institute on Minority Health and Health Disparities (NIMHD) Award Number U54MD000538, and the preparation of this manuscript was supported in part by U.S. Department of Health and Human Services, Centers for Disease Control and Prevention (CDC) Award Numbers NU38OT2020001477, CFDA number 93.421, 1NH23IP922639-01-00, CFDA number 93.185, and NIH NHLBI Community Engagement Alliance (CEAL) Non-Federal 1OT2HL156812-01, Westat Sub-OTA No: 6793-02-S013. LÐ was supported by the NIH Resource Centers for Minority Aging Research (RCMAR) Award Number 5P30AG059302. The contents of this publication are solely the responsibility of the authors and do not necessarily represent the official views of the funders.

## Author Disclaimer

The contents of this publication are solely the responsibility of the authors and do not necessarily represent the official views of the funders.

## Conflict of Interest

The authors declare that the research was conducted in the absence of any commercial or financial relationships that could be construed as a potential conflict of interest.

## Publisher's Note

All claims expressed in this article are solely those of the authors and do not necessarily represent those of their affiliated organizations, or those of the publisher, the editors and the reviewers. Any product that may be evaluated in this article, or claim that may be made by its manufacturer, is not guaranteed or endorsed by the publisher.

## Footnotes

^1^Mohsin F, Ali SH, Parikh R, Chong SK, DiClemente RJ. Social media use and family interaction among Asian American young adults: identifying disparities to inform interpersonal health interventions.
